# Perceived fear of COVID-19 and its associated factors among Nepalese older adults in eastern Nepal: A cross-sectional study

**DOI:** 10.1371/journal.pone.0254825

**Published:** 2021-07-26

**Authors:** Uday Narayan Yadav, Om Prakash Yadav, Devendra Raj Singh, Saruna Ghimire, Binod Rayamajhee, Sabuj Kanti Mistry, Lal Bahadur Rawal, ARM Mehrab Ali, Man Kumar Tamang, Suresh Mehta

**Affiliations:** 1 Centre for Primary Health Care and Equity, UNSW, Sydney, Australia; 2 School of Population Health, UNSW, Sydney, Australia; 3 Centre for Research, Policy and Implementation, Biratnagar, Nepal; 4 Torrens University, Sydney, Australia; 5 School of Health Medical and Social Sciences, Central Queensland University, Sydney, Australia; 6 Ministry of Health and Population, Kathmandu, Nepal; 7 Department of Public Health, Asian College for Advance Studies, Purbanchal University, Biratnagar, Nepal; 8 Department of Sociology and Gerontology and Scripps Gerontology Center, Miami University, Oxford, OH, United States of America; 9 School of Optometry and Vision Science, Faculty of Medicine and Health Sciences, UNSW, Sydney, Australia; 10 Department of Infection and Immunology, Kathmandu Research Institute for Biological Sciences, Lalitpur, Nepal; 11 BRAC University, Dhaka, Bangladesh; 12 Aureolin Research, Consultancy and Expertise Development Foundation, Dhaka, Bangladesh; 13 Queensland Brain Institute, The University of Queensland, Brisbane, Australia; University of Tokyo, JAPAN

## Abstract

**Background:**

Coronavirus disease 2019 (COVID-19) has affected all age groups worldwide, but older adults have been affected greatly with an increased risk of severe illness and mortality. Nepal is struggling with the COVID-19 pandemic. The normal life of older adults, one of the vulnerable populations to COVID-19 infection, has been primarily impacted. The current evidence shows that the COVID-19 virus strains are deadly, and non-compliance to standard protocols can have serious consequences, increasing fear among older adults. This study assessed the perceived fear of COVID-19 and associated factors among older adults in eastern Nepal.

**Methods:**

A cross-sectional study was conducted between July and September 2020 among 847 older adults (≥60 years) residing in three districts of eastern Nepal. Perceived fear of COVID-19 was measured using the seven-item Fear of COVID-19 Scale (FCV-19S). Multivariate logistic regression identified the factors associated with COVID-19 fear.

**Results:**

The mean score of the FCV-19S was 18.1 (SD = 5.2), and a sizeable proportion of older adults, ranging between 12%-34%, agreed with the seven items of the fear scale. Increasing age, Dalit ethnicity, remoteness to the health facility, and being concerned or overwhelmed with the COVID-19 were associated with greater fear of COVID-19. In contrast, preexisting health conditions were inversely associated with fear.

**Conclusion:**

Greater fear of the COVID-19 among the older adults in eastern Nepal suggests that during unprecedented times such as the current pandemic, the psychological needs of older adults should be prioritized. Establishing and integrating community-level mental health support as a part of the COVID-19 preparedness and response plan might help to combat COVID-19 fear among them.

## Background

As of June 13, 2021, over 175 million confirmed cases and over 3.7 million deaths are reported worldwide from SARS-CoV-2 infection (COVID-19) [1]. By the same date, Nepal reported 284,673 confirmed cases and 3,083 deaths from COVID-19 [2]. The ongoing COVID-19 pandemic has been the center of focus globally and covered extensively in local and international news outlets. Much of the global and regional focus has been on the prevention of transmission and therapeutics, which are undoubtedly pressing public health needs during a pandemic, mental health issues have been less emphasized. Studies have reported a higher prevalence (25% to 90%) of mental illness such as depression, anxiety, psychosis, and stress symptoms among aged people in Nepal even in the pre-COVID-19 era [3, 4].

The life of older adults has been disrupted in most parts of the world, and anxiety is common among people to accept the increasing cruelty of COVID-19. Although most people seem to be physically, mentally, socio-economically impacted by the pandemic, the impact may be greater for certain groups such as the older adults who are at an elevated risk of severe illness and mortality [5]; the COVID-19 fatality rate for those over 80 years of age is five times the global average [6]. Given the increased rate of infection and mortality, it is evident that older adults are at risk of fear and worry due to the current unprecedented pandemic condition. Fear may affect older adults’ feelings, mood, or behavior, impacting their ability to function physically, socially, and cognitively each day [7, 8]. COVID-19 has exacerbated the situation of older adults by escalating the feeling of fear, panic/great worry due to overthinking of COVID-19 infection, death, or isolation from home/family [9]. In addition, feelings of loneliness, uneasiness, uncertainty, financial impact, preexisting diseases, limited physical activity, and increased smoking are the reported factors associated with higher levels of fear and psychological distress among older adults during COVID-19 [10–12].

The Senior Citizen Act of Nepal defines individuals aged ≥60 years as senior citizens or older adults [13]. The average life expectancy at birth in Nepal has increased from 49.6 years in 1981 to 70.6 years in 2016 [14], resulting in a burgeoning population of older adults. According to recent estimates for 2019, there were 2.67 million older adults in Nepal, representing 8.6% of the total population [15]. The growth rate for older adults is 3.5% per year, greater than the national growth rate of 1.35% [16]. Evidence from severe acute respiratory syndrome and the COVID-19 outbreak showed that stress, anxiety, and other psychiatric morbidities lead to fear [17].

There is scarce data on fear and its predictors among older adults in south Asian countries, mainly from resource-limited countries like Nepal. To the best of our knowledge, this is the first study ever been conducted to assess the fear of COVID-19 and its associated factors among the older adults at the community settings in Nepal amid the COVID-19 pandemic.

## Materials and methods

### Study design and participants

**Fig 1:**
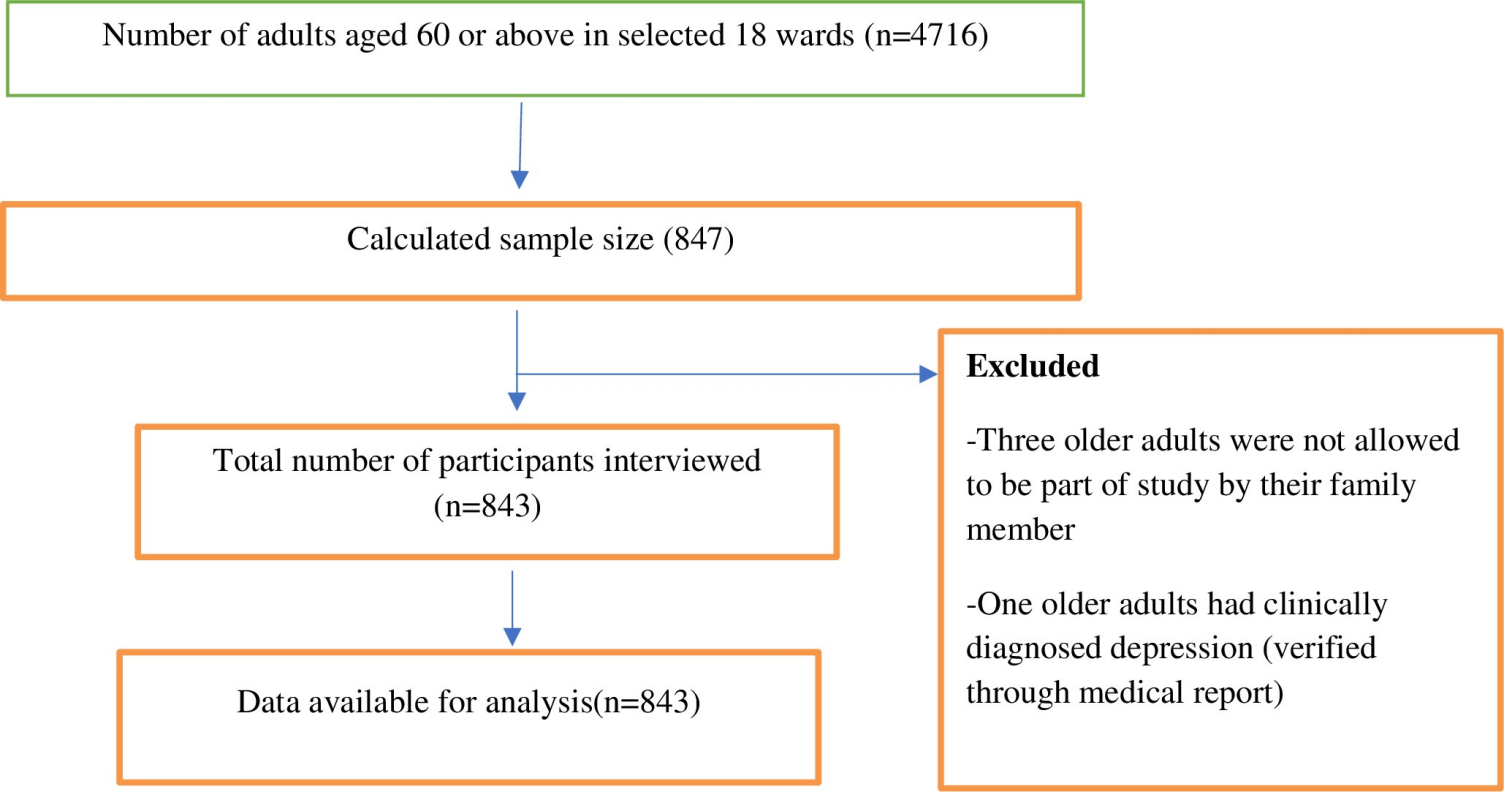
Flow diagram illustrating recruitment of study subjects based on the Strengthening the Reporting of Observational Studies in Epidemiology (STROBE) guidelines.

A cross-sectional study was conducted between July and September 2020 among the older adults (≥60 years) residing in three districts (*Morang*, *Pachthar*, and *Terathum*) of Province 1 in eastern Nepal. *Morang* district is located in the plains, while *Pachthar* and *Terathum* in a hilly region of Nepal. The sample size of 847 was calculated considering the following parameters: unknown prevalence  =  50%, CI  =  95.0%, sampling error  =  5.0%, design effect  =  2, and non-response rate  =  5.0%. Multi-stage cluster sampling recruited older adults (**Fig 1**). In the first stage, three districts from Province 1 were selected randomly. Then, one urban and one rural municipality in each district were selected randomly. Subsequently, from each municipality, three wards (lowest administrative units in Nepal) were randomly selected. A proportionate simple random sampling technique was used to recruit older adults from each ward in the final step. Representatives from the municipalities provided the list of eligible older adults in the selected wards. Eligibility criteria included age ≥60 years, Nepali nationals with a minimum of one year of residence in the community. The exclusion criteria included older adults residing in nursing care, having any mental conditions, hearing disability, or inability to communicate.

### Data collection and study variables

Data were collected by semi-structured interviews using a validated survey questionnaire [18]. Surveyors were 12 trained health care providers with proficiency certificate in General Medicine (three years course) working for the government of Nepal in the selected districts. Prior to data collection, surveyors were trained through the zoom meeting facilitated by five authors (UNY, OPY, DRS, SKM, and SM) for four hours with 30 minutes of break. Two zoom sessions were conducted to capacitate the enumerators. Surveyors were trained on study tools, participant recruitment, ethical aspects, and data collection techniques. Surveyors visited the households and collected the data using the KoBo Toolbox mobile app [19]. Moreover, a Whatsapp group was created and shared with enumerators to troubleshoot problems during the data collection process.

### Dependent variable measurement

COVID-19 fear was the primary outcome measured using the seven-item Fear of COVID-19 Scale (FCV-19S) developed and validated by Ahorus et al. among the general Iranian population [18]. Older adult’s agreement/disagreement with the seven items was assessed using a five-point Likert scale (ranging from 1 = “strongly disagree,” 3 = “neither agree nor disagree,” and 5 = “strongly agree”). Hence, the cumulative score ranged from 7 to 35, where the higher the scores, the greater the fear of COVID-19.

The internal consistency or the reliability of the scale among Nepali older adults was acceptable (Cronbach’s α = 0.86, McDonald’s ω = 0.88 and Guttmann’s λ = 0.90). Confirmatory factor analysis (CFA) was conducted to validate the scale among study participants where the standardized root mean square residual (SRMR) close to 0.06, and the Goodness of Fit Index (GFI), Normed Fit Index (NFI) and Comparative Fit Index (CFI) close to 0.90 were considered to be acceptable [20]. In the CFA, the scale indices were well fit and within the acceptable limit [χ2 (14, N = 843) = 345.82, p < 0.01; SRMR = 0.062; GFI = 0.884; NFI = 0 .917; IFI = 0.920; CFI = 0.920].

### Independent variable measurement

Independent variables included age group, gender, marital status, family type, ethnicity, education, occupation, urban/rural residence, walking distance to the nearest health facility, any preexisting chronic health problems, any current medications, financial hardships with health care access, the need of additional care from family members/caregivers, recipient of social security allowance, source of COVID-19 information, and feeling overwhelmed and concerned because of COVID-19 pandemic. These co-variates definitions and measurements are described in our previous work [21, 22]. For the variable “sources of COVID-19 information”, in multiple response questions, older adults were asked to list their source of information for COVID-19. Older adults were also asked, “how overwhelmed are you feeling in this *COVID-19* (Not at all/Somewhat overwhelmed/ Very overwhelmed)” and “how concerned are you because of this *COVID-19* (Not at all/Somewhat concerned/, Very concerned)”.

The English version of the questionnaires was first translated to Nepali and then back-translated to English by three researchers to ensure the contents’ consistency. Piloting the questionnaires among a small sample (n = 10) of older adults helped refine the final version by considering their remarks from the older adults. Following piloting, minor editorial issues were resolved, such as “COVID-19” was replaced with the term coronavirus throughout, and the question that aimed to capture proximity to the health facility was clarified by specifying “walking distance to the nearest health facility.”

### Ethics

This study received ethics approval (Ref# 150/2020) from the ethics board of Nepal Health Research Council (NHRC), Kathmandu, Nepal. All study participants provided informed written consent. For participants unable to read and write, proxy written consent was obtained from their close guardians. Participation was voluntary, and older adults did not receive any financial compensation. After completing the interview, community health care providers delivered 10 minutes of counseling services on dealing with COVID-19 was delivered to all participants.

### Statistical analyses

**Fig 2:**
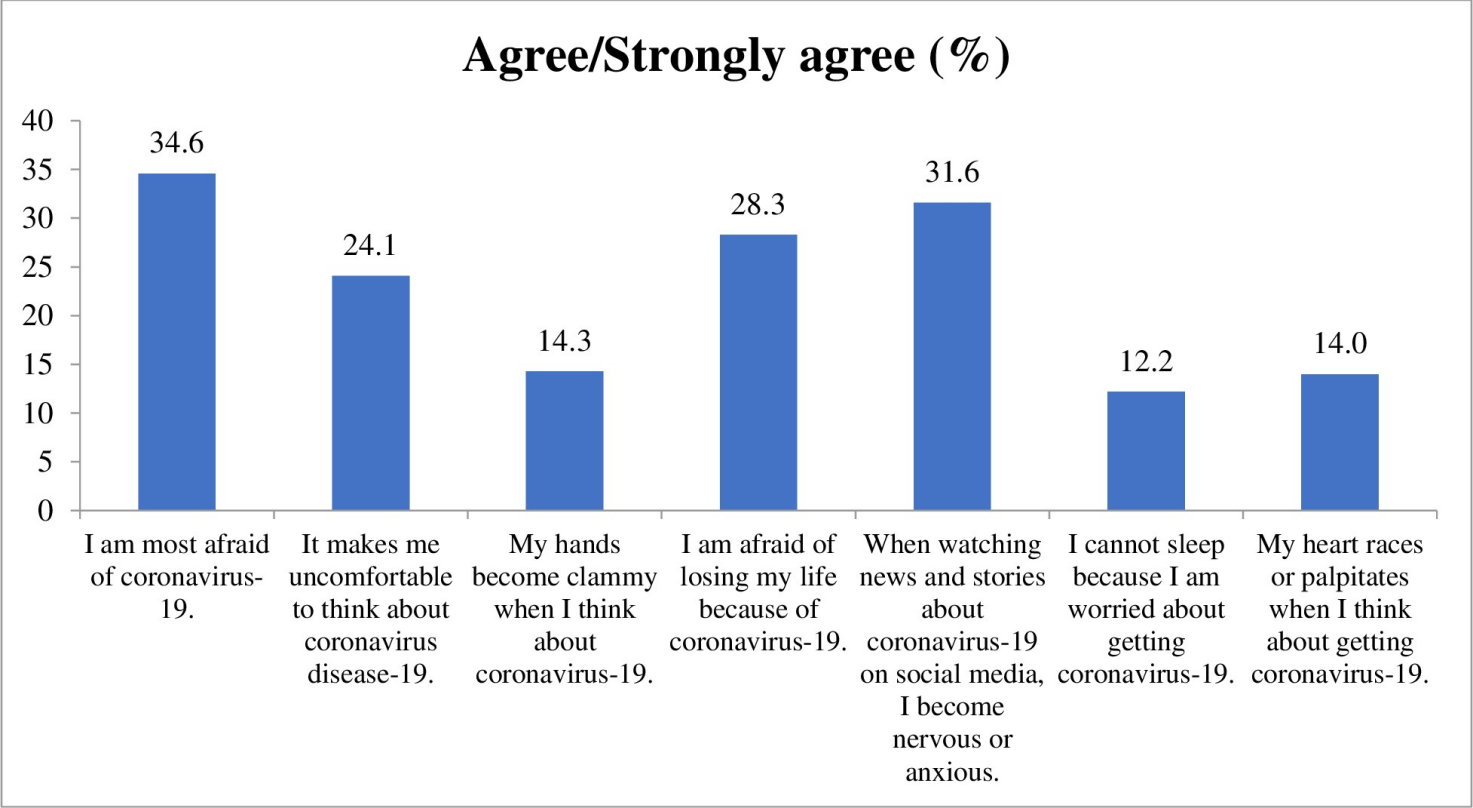
Older adults’ agreement on the seven items of the COVID-19 fear scale.

Mean (SD) and frequency (%) is used to depict participants’ characteristics. Independent t-tests and ANOVA evaluated the mean differences in the FCV-19S score by participants’ characteristics. The bar graph (**Fig 2**) shows the older adults’ agreement with the seven items of FCV-19S. A generalized estimating equation, adjusting the sampling design, was used to examine the factors associated with the COVID 19 fear. We assessed the multicollinearity of covariates using Variance Inflation Factors (VIFs). The VIFs for all covariates that were included in the logistic regression analysis were less than 2.0. The variables with p<0.2 in the bivariate analyses were included in the final multivariate model. STATA 15 and JASP 0.13.1 were used for data analyses.

## Results

**Table 1** describes older adults’ characteristics and their bivariate association with the COVID-19 fear score. Among the 843 older adults, 45% were aged 60–69 years, 49% were female, 76% were married, and 87% lived in joint/extended families. Most of the participants had no formal education (68%), had preexisting conditions (64%) or used medications (51%), and had financial hardships to access health care (55%) (**Table 1**).

**Table 1:** Participant’s characteristics and COVID-19 fear using bivariate analysis.

		Frequency	%	COVID-19 fear score		
				Mean	SD	P
Total		843	100.0	18.1	5.2	
**Age (Years)**						
	60–69	383	45.4	16.7	3.6	<0.001
	70–79	315	37.4	19.1	6.1	
	80–89	123	14.6	19.5	5.9	
	≥90	22	2.6	20.5	5.1	
**Gender**						
	Female	412	48.9	18.2	5.3	0.782
	Male	431	51.1	18.1	5.1	
**Marital status**						
	Married	639	75.8	17.8	5.3	0.005
	^1^Without partner	204	24.2	19.0	5.0	
**Family type**						
	Nuclear	111	13.2	18.2	4.1	0.908
	Joint/extended	732	86.8	18.1	5.4	
**Ethnicity**						
	Brahmins/Chhetris	266	31.6	18.8	5.4	
	Dalits	50	5.9	19.9	5.3	<0.001
	Madhesi	132	15.7	15.7	3.0	
	Indigenous	276	32.7	19.0	6.0	
	Others	119	14.1	16.2	3.1	
**Education**						
	No formal education	577	68.4	17.8	5.1	0.011
	Formally schooled	266	31.6	18.8	5.4	
**Occupation**						
	Retired	51	6.0	17.4	3.7	<0.001
	Agriculture	400	47.4	19.4	5.9	
	Business	34	4.0	18.2	4.7	
	Housewife	223	26.5	17.0	4.3	
	Wages based labors	39	4.6	17.4	4.1	
	Others (plumbers, service holders and carpenters)	96	11.4	15.9	3.6	
**Residence**						
	Rural	370	43.9	18.6	6.0	0.012
	Urban	473	56.1	17.7	4.4	
**Walking distance to the nearest health facility**						
	<30 mins	272	32.3	16.7	4.8	<0.001
	30–60 mins	372	44.1	18.2	4.7	
	>60 mins	199	23.6	19.7	6.1	
**Preexisting health conditions**^**2**^						
	No	301	35.7	19.4	6.4	<0.001
	Yes	542	64.3	17.4	4.2	
**Current medications use**						
	No	409	48.5	18.6	5.9	0.005
	Yes	433	51.4	17.6	4.4	
**Financial hardships with health care**						
	No/not sure	379	45.0	18.7	6.5	0.001
	Yes	464	55.0	17.5	3.8	
**Need additional care from family**						
	No	320	38.0	17.1	4.7	<0.001
	Yes	523	62.0	18.7	5.4	
**Receiving social security allowance**						
	No	409	48.5	17.1	4.6	<0.001
	Yes	434	51.5	19.0	5.6	
**Source of information of COVID-19**^3^						
	Family/relatives	732	85.8	18.2	5.0	0.439
	TV/Radio	672	79.7	18.5	5.5	<0.001
	Newspapers	58	6.9	17.3	4.1	0.242
	Social medias	88	10.4	17.6	6.2	0.302
	Health workers	80	9.5	19.5	5.8	0.011
	Internet (e.g. Online newspaper and google search)	20	2.4	16.4	2.5	0.129
	Others	3	0.4	11.7	4.0	0.032
**Concerned about COVID-19 pandemic**						
	Not at all	337	40.0	15.4	3.4	<0.001
	Somewhat concerned	452	53.6	19.6	5.4	
	Very concerned	54	6.4	22.5	4.8	
**Feeling overwhelmed with the COVID-19 pandemic**						
	No/not sure	656	77.8	17.8	5.2	<0.001
	Yes	99	11.7	20.7	5.5	

^1^Includes widow/widower/separated/never married.

^2^Includes diabetes, hypertension, cardiovascular disease, chronic kidney disease, respiratory disease (COPD and Asthma), and osteoarthritis.

^3^Multiple responses.

The mean score of the FCV-19S was 18.1 (SD 5.2) (**Table 1**). Older adults’ agreement on the seven items of FCV-19S is shown in **Fig 2**, whereby 34.6%, 24.1%, 14.3%, 28.0%, 31.6%, 12.2%, and 14.0% participants agreed being afraid, uncomfortable, clamminess, fearful of losing life, being nervous or anxious, and experienced sleeplessness and palpitation due to COVID-19, respectively. Mean differences in fear of COVID-19 were noted for several independent variables (p<0.05), including age category, marital status, ethnicity, education, occupation, residence, distance to the nearest health facility, preexisting health conditions, medications use, financial hardships with health care, needing additional care from family, receiving social security allowance, and being concerned or overwhelmed with the COVID-19 pandemic (**Table 1**).

### Factors associated with the fear of COVID-19 among Nepali older adults

**Table 2** presents factors associated with the fear of COVID-19 among older adults. Increasing age, Dalit ethnicity, remoteness to the health facility, and being concerned or overwhelmed with the COVID-19 pandemic were associated with greater fear of COVID-19. Preexisting health conditions among older adults were inversely related to fear of COVID-19.

**Table 2:** Factors associated with the fear of COVID-19 among Nepali older adults (n = 843).

		Β Coefficient	Std. Err.	P-value	95% CI	
					Lower	Upper
**Age (Years)**						
	60–69	Ref.				
	70–79	1.75	0.98	0.102	-0.41	3.92
	80–89	2.65	1.01	0.024	0.43	4.88
	>90	3.69	1.51	0.032	0.38	7.01
**Marital status**						
	Married	Ref.				
	^2^Without partner	0.62	0.36	0.116	-0.18	1.42
**Ethnicity**						
	Brahmins/Chhetris	Ref.				
	Dalits	1.43	0.32	0.001	0.72	2.14
	Madhesi	-1.24	0.82	0.161	-3.05	0.57
	Indigenous	-0.09	0.57	0.874	-1.35	1.16
	Others	-2.16	1.54	0.190	-5.55	1.24
**Education**						
	No formal education	Ref.				
	Formally schooled	0.05	0.34	0.893	-0.71	0.80
**Occupation**						
	Retired	Ref.				
	Agriculture	1.12	0.69	0.134	-0.41	2.65
	Business	0.87	1.25	0.503	-1.89	3.63
	Housewife	0.07	0.83	0.933	-1.76	1.91
	Wages based labors	1.23	1.12	0.294	-1.23	3.69
	Others	-0.16	1.09	0.884	-2.56	2.23
**Residence**						
	Rural	Ref.				
	Urban	-2.35	1.71	0.196	-6.12	1.41
**Walking distance to the nearest health facility**						
	<30 mins	Ref.				
	30–60 mins	0.72	0.27	0.023	0.12	1.33
	>60 mins	1.65	0.45	0.004	0.65	2.65
**Preexisting health conditions **						
	No	Ref.				
	Yes	-1.94	0.83	0.039	-3.76	-0.12
**Current medications use**						
	No	Ref.				
	Yes	-0.19	0.68	0.780	-1.68	1.30
**Financial hardships with health care**						
	No/not sure	Ref.				
	Yes	-0.26	0.88	0.776	-2.19	1.68
**Need additional care from family**						
	No	Ref.				
	Yes	0.77	0.72	0.306	-0.81	2.34
**Receiving social security allowance**						
	No	Ref.				
	Yes	-0.10	0.49	0.850	-1.18	0.99
**Source of information of COVID-19 (Ref. = No)**						
	TV/Radio	1.46	0.73	0.070	-0.14	3.06
	Health workers	0.63	0.47	0.202	-0.39	1.66
	Others (information gathered from social media, internet and family/relatives)	-3.56	1.32	0.021	-6.46	-0.66
**Concerned about COVID-19 pandemic**						
	Not at all	Ref.				
	Somewhat concerned	2.95	0.70	0.001	1.42	4.48
	Very concerned	6.02	0.51	<0.001	4.91	7.13
**Feeling overwhelmed with the COVID-19 pandemic**						
	No/not sure	Ref.				
	Yes	1.88	0.61	0.011	0.53	3.23

Compared to the youngest age group, there was a gradual increase in the fear coefficient with the age categories suggesting that the oldest adults were the most fearful. Notably, the coefficient was not statistically significant for the age group 70–79. Compared to Brahmins/Chhetris, the Dalits ethnic group had a higher fear level (Coef.:1.43; 95%CI: 0.72 to 2.14). Likewise, a dose-response relationship was observed between perceived COVID-19 fear and the proximity or remoteness to the nearest health facility, measured in terms of approximate walking time to the facility; those at the most remoteness (>60 mins walking time) had the highest fear coefficient (Coef. 1.65; 95%CI: 0.65 to 2.65) compared to those at the proximity (<30 mins walking time). Older adults with preexisting health conditions were less fearful of COVID-19 than those without any conditions (Coef.: -1.94; 95%CI: -3.76 to -0.12). Moreover, those who were concerned about COVID-19 [(somewhat concerned coef.: 2.95; 95%CI: 1.42 to 4.48) and (very concerned coef.: 6.02; 95%CI: 4.91 to 7.13)] were more fearful than those who were not concerned at all. Likewise, older adults overwhelmed with the COVID-19 pandemic were more fearful than their counterparts who were not overwhelmed with the COVID-19 pandemic (Coef.: 1.88; 95%CI: 0.53 to 3.23).

In the adjusted model, the coefficient for the variable’s marital status, education level, occupation, residence, current medications use, financial hardships with health care, needing additional care from family, and receiving social security allowance lost statistical significance after adjusting for covariates (**Table 2**).

## Discussion

In the current research, older adults living in eastern Nepal experienced different levels of COVID-19 fear on the seven items of the used fear scale. A sizable proportion of older adults reported an agreement (ranging between 12%-34%) on the seven items of FCV-19S. Although the literature on fear of COVID-19 among older adults lacks from Nepal, experts opined that COVID-19 has heavily affected older adults’ mental health as they are more susceptible to fear and anxiety problems during the pandemic [23, 24]. While there is scarce evidence on COVID 19 fear among Nepalese older adults, our findings align with the recently published evidence that reported fear, psychosocial effects, and uncertainty due to COVID-19 in Nepal and other settings [25–28]. A recent study from Bangladesh reported a higher COVID-19 fear among Bangladeshi older adults [26]. Variations in older adult’s responses on items of fear scale might differ across settings because of different levels of emotional responses to the phenomenology of the pandemic, such as desist warnings, unending uncertainty, and concern over people’s death amid COVID [29, 30].

In the current study, increased age was associated with greater fear of COVID-19, which was plausible given that the impact of COVID-19 in terms of hospitalizations, ICU admissions, and fatality rate, is higher among older adults [5, 6, 31]. Rapid spread of COVID-19, extensive news coverage portraying older adults’ vulnerability to the infection coupled with COVID-19 misinformation and challenges to access health care may explain why older adults were more fearful. However, in contrast to our findings, a study conducted in Bangladesh did not find an association between older adults’ age and fear of COVID-19 [26]. This discrepancy could be because of differences in study settings and small number oldest age category in the Bangladeshi study. Our findings suggest the need to pay more attention to the mental health needs of older adults within a higher age bracket.

Another notable finding from this study was the greater perceived fear among Dalits (low caste ethnic minority as per traditional Hindu caste system) compared to the Brahmin/Chhetris or so-called upper caste. Notably, the Dalit ethnic group was historically considered “untouchables” until the recent past and is still disadvantaged in terms of opportunities. The role of social determinants of health and their linkage to poor health outcomes among minorities is well documented [32]. Although the socioeconomic and health inequalities between the upper caste group and Dalits have existed historically, even in the pre-COVID-19 era, the current pandemic, disproportionally affecting marginalized communities [33], has led us to rethink and revisit the disparities by ethnicity. Thus, the greater perceived fear of COVID-19 among older adults belonging to Dalit ethnic groups could be attributed to the relative disadvantages in terms of access to health care and other resources.

This study found that being distant from health facilities (i.e., remoteness to the health facility) was associated with greater fear of COVID-19. The possible explanation could be that most local healthcare facilities have halted their services due to fear of COVID-19 transmission [34]. Moreover, older adults who reside farther away from the health facility may think it would be difficult to reach health facilities/centers for testing or COVID-19 related health care on time, resulting in fear development among participants.

Our study identified that older adults overwhelmed and concerned about the effect of the COVID-19 were more fearful than those who were indifferent to it. This is anticipated as fear of COVID-19 cripples when people become more concerned about its lethal outcomes, triggering psychological distress [35]. Poor health literacy among older adults in Nepal, specifically the oldest age groups [36], may avert their ability to access, analyze, and appraise the information on COVID-19. Furthermore, a meta-analysis on aging and the misinformation effect found that older adults aged above 65 are more vulnerable to misinformation [37]. Misinformation could aggravate fear and increase the sense of helplessness [38]. Misinformation has prevailed during the current pandemic, and reports of older adults perceiving that COVID-19 had periled their existence [35] is an example that emphasizes the negative mental health impact of COVID-19 among the most vulnerable group. Such misinformation and ageism may make older adults very concerned and overwhelmed about the pandemic, which eventually can accumulate stress, fear, panic, and depression [39]. Nevertheless, we have not measured COVID-19 related misinformation, so the interpretation of this explanation needs to be made with caution. Moreover, subsyndromal mental health (a depressive state having two or more symptoms of depression such as insomnia, feeling tired all the time, trouble concentrating, slowed thinking, etc.) consequences might have spiked among the older adults due to isolation and loneliness [40–42]. It is pertinent to note that this study did not assess subsyndromal mental health conditions, so one should be cautious while interpreting our explanation.

The current study did not find the association between sources of information, such as social media, newspapers, and COVID-19 fear. The possible explanation could be that older adults with limited literacy or no formal education are less likely to follow pandemic-related news, information, or social media. In contrast to current findings, emerging evidence has shown that mass media, including social media, plays a pivotal role in disseminating information on COVID-19 infection and death rates, making people more concerned, resulting in adverse psychological effects [43, 44].

Our finding contrasts with another study that reported that older adults with preexisting conditions were more concerned about contracting COVID-19 [45]. This could be because older adults with preexisting conditions have already navigated health services, and thus they know where and how to access health care [46]. Supporting our findings, a recent study from Nepal reported that people with multiple chronic conditions had a good level of self-management skills, where we can argue that people with good self-management skills might have less fear [46].

One of the strengths of this study is that it is the first of its kind to assess the level of COVID-19 related fear among older adults in Nepal. Another strength was that this study used the Nepali version of the survey instrument validated with ten target participants to check if the target population correctly chose the included questions’ responses. Moreover, questions framed in a positive or neutral light helped solicit truthful answers from the study participants. These strategies may have reduced the social-desirability bias in this study. Despite these strategies, we acknowledge that social-desirability bias could have occurred as we relied on the information provided by participants. As with other studies, this study does have some limitations. First, the study was conducted in three districts of Province 1, limiting the generalizability to other provinces and/or settings of Nepal. Second, it is worthy of mentioning that the status of fear might have altered after COVID-19 cases continue to increase in Nepal. Lastly, the FCV-19S has not been validated in Nepal yet; therefore, the stability of the scale over time must be examined.

## Conclusion

The greater fear of the COVID-19 pandemic among the older adults suggests that during unprecedented times such as the current pandemic, due care of the psychological needs of the older adults in eastern Nepal need to be taken. Thus, addressing the mental health needs of older adults should be one of the priorities of pandemic management. In this overwhelming condition, social connections are extremely important for maintaining older adults’ physical and mental wellbeing. Therefore, it is important to engage family members and friends through phone calls or video chats to cultivate social connections and promote healthy relationships with others. Thus, fostering meaningful connections and creating a sense of belonging will enhance motivation and reduce the fear and risk of COVID-19. Moreover, community health workers for the same community can be trained virtually on a wellbeing support package to provide psychological support following World Health Organization’s COVID-19 safety protocols that may improve older adults’ psychological wellbeing.

## Supporting information

S1 Dataset (SAV)
